# Hepatitis E Virus Infections: Epidemiology, Genetic Diversity, and Clinical Considerations

**DOI:** 10.3390/v15061389

**Published:** 2023-06-17

**Authors:** Busara Songtanin, Adebayo J. Molehin, Kevin Brittan, Wuttiporn Manatsathit, Kenneth Nugent

**Affiliations:** 1 Department of Internal Medicine, Texas Tech University Health Sciences Center, Lubbock, TX 79430, USA; busara.songtanin@ttuhsc.edu; 2 Department of Microbiology & Immunology, College of Graduate Studies, Midwestern University, Glendale, AZ 85308, USA; amoleh@midwestern.edu; 3 Department of Internal Medicine, University of Nebraska Medical Center, Omaha, NE 68198, USA; kbrittan@unmc.edu; 4 Department of Gastroenterology and Hepatology, University of Nebraska Medical Center, Omaha, NE 68198, USA; shane.manatsathit@unmc.edu

**Keywords:** hepatitis E virus, hepatitis E infection, HEV epidemiology, HEV diagnosis, clinical manifestation, organ transplantation

## Abstract

According to the World Health Organization, approximately 20 million people worldwide are infected annually with the hepatitis E virus (HEV). There are four main genotypes of HEV. Genotype 1 and genotype 2 are common in developing countries and are transmitted by contaminated water from a fecal–oral route. Genotype 3 and genotype 4 are common in developed countries and can lead to occasional transmission to humans via undercooked meat. Hepatitis E virus 1 and HEV3 can lead to fulminant hepatitis, and HEV3 can lead to chronic hepatitis and cirrhosis in immunocompromised patients. The majority of patients with HEV infection are asymptomatic and usually have spontaneous viral clearance without treatment. However, infection in immunocompromised individuals can lead to chronic HEV infection. Both acute and chronic HEV infections can have extrahepatic manifestations. No specific treatment is required for acute HEV infection, no treatment has been approved in chronic infection, and no HEV vaccine has been approved by the (United States) Food and Drug Administration. This review focuses on the molecular virology (HEV life cycle, genotypes, model systems, zoonosis), pathogenesis, clinical manifestation, and treatment of chronic HEV infection, especially in immunocompromised patients, to provide clinicians a better understanding of the global distribution of these infections and the significant effect they can have on immunocompromised patients.

## 1. Introduction

A recent meta-analysis reported that 12.5% of the worldwide population is infected with the hepatitis E virus (HEV) during their lifetimes based on positive anti-HEV immunoglobulin G (IgG) antibody tests [1]. The highest prevalence of HEV infection occurs in Africa followed by Asia, Europe, North America, and South America. Hepatitis E virus is an emerging zoonotic infection, common in low- to middle-income countries [2], causing 3.3% of the deaths associated with viral infections [2]. Genotypes 1 and 2 are more common in East Asian countries, with transmission occurring from human to human from a fecal–oral route, while genotypes 3 and 4 are more common in Europe and America (North and South) and transmitted from a zoonotic infection, most commonly undercooked meat [1,2]. The majority of HEV-infected patients are asymptomatic, with a ratio of symptomatic to asymptotic presentations of 1:2 to 1:13 [3]. Most cases of acute hepatitis are self-limited and do not require treatment, some patients develop fulminant hepatitis, and some develop chronic hepatitis. Fulminant hepatitis occurs more frequently in pregnant women. In addition to hepatic disorders, patients can develop extrahepatic complications. Progression of the disease to chronic infection occurs more frequently in immunocompromised patients with solid-organ transplantation, hematologic malignancy, HIV infection, and chronic immunosuppression with rheumatic disorders. Currently, the first-line treatment is ribavirin after reduction of immunosuppressive therapy, if possible [4]. In patients who fail ribavirin treatment, pegylated interferon-alpha should be considered.

Many clinicians working in developed countries may not be familiar with the medical problems associated with hepatitis E infections and may not suspect this virus as a cause of undiagnosed hepatitis. This review highlights the discovery, epidemiology, and general aspects of hepatitis E virus genetic diversity. Furthermore, it provides some insights into the HEV life cycle and clinical presentations in both immunocompetent and immunocompromised patients to underscore the need for more research into HEV pathophysiology. Last, this review describes the currently available HEV diagnostic techniques and treatment options.

## 2. Epidemiology of Hepatitis E Virus

Hepatitis E virus is the fifth known cause of human viral hepatitis and is probably the most common etiologic agent of acute hepatitis and jaundice globally [5,6]. Following its initial identification in the 1980s as non-A and non-B hepatitis, this infectious waterborne disease that was prevalent in developing regions but rare in industrialized nations [7] caused by the hepatitis E virus (HEV) was later identified using immune electron microscopy, and the viral genome was determined in 1990 [8]. There are 20 million HEV infections throughout the world annually with over 3 million symptomatic cases and approximately 44,000 deaths [9]. In developing countries with poor sanitation, HEV transmission is primarily through the fecal–oral route after drinking water contaminated with human feces; sporadic and cluster cases of hepatitis E infections in industrialized countries are associated with the ingestion of raw or undercooked meat products [10]. Due to HEV’s high stability against drying, transmission should also be expected on surfaces after contact with contaminated meat or excretions from infected hosts [11]. Acute HEV infections usually do not progress to acute liver failure or chronic infections, but pregnant women have a high risk of developing fulminant hepatic failure with a case-fatality rate of up to 30% [12]. Furthermore, HEV infections in immunosuppressed patients can cause chronic infections leading to cirrhosis that requires transplantation if left untreated [13]. The discovery of zoonotic HEV from domestic pigs in the late 1990s in the United States [14] led to HEV being recognized as an important emerging zoonotic pathogen with a wide range of reservoirs, including deer, rabbits, camels, and rats. In addition, more strains of HEV have been identified in several animal species, with their host range and pathogenicity under investigation [15].

## 3. Characteristics and Genetic Diversity of Hepatitis E Virus

**Figure 1:**
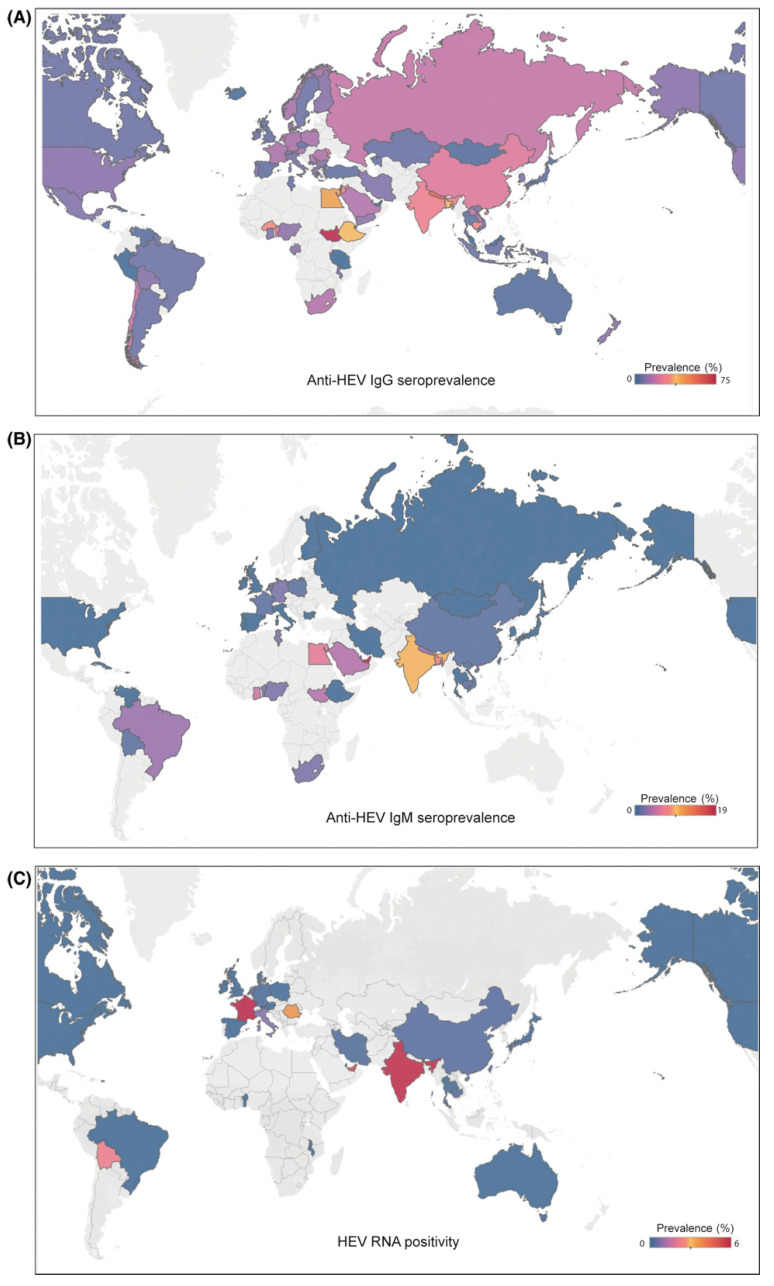
Distribution of hepatitis E based on IgG seroprevalence (**A**), IgM seroprevalence (**B**), and hepatitis E RNA positivity (**C**) (adapted from Li et al. [1] with permission).

**Figure 2:**
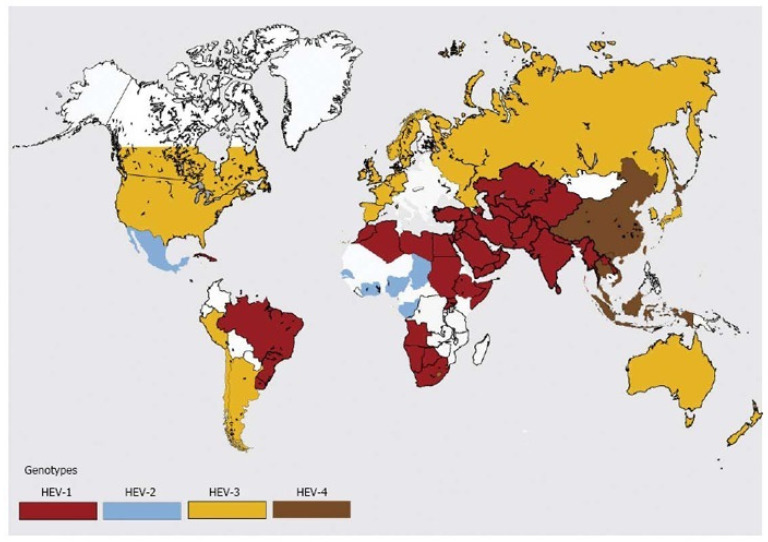
Distribution of hepatitis E virus genotypes (adapted from Khuroo et al. [23] with permission).

Hepatitis E virus belongs to the family Hepeviridae, which consists of two genera, namely *Orthohepevirus* and *Piscihepevirus*. There are four species of *Orthohepevirus* (*Orthohepevirus A* to *D*) and one species of *Piscihepevirus* (*Piscihepevirus A*) [16]. *Orthohepevirus A* is divided into at least eight distinct genotypes with genotypes 1 and 2 responsible for human infections, causing significant waterborne outbreaks in endemic regions of South and Southeast Asia, Africa, and Mexico. Genotypes 3 and 4 are responsible for sporadic cases of hepatitis E in Europe and East Asia, where they infect a wide range of mammals, such as swine, deer, rabbits, and humans (Figure 1 and Figure 2). Genotypes 5 and 6 were identified in wild boars in Japan; genotype 7 and genotype 8 were identified in dromedaries and Bactrian camels in Middle Eastern countries and China, respectively. *Orthohepevirus B* and *D* infect birds and bats, respectively; *Orthohepevirus C* infects rats, shrews, ferrets, minks, and wild rodents [17]. The primary sources of human zoonotic infections are genotypes 3 and 4, but other animal strains, including genotypes 5, 7, and 8 and rat HEVs from species of *Orthohepevirus* C, also have zoonotic potential [15,18]. With the ever-growing host range and identification of genetically divergent HEV strains made possible through advancing technologies, the taxonomy of the family *Hepeviridae* will undoubtedly continue to evolve. 

The genome of HEV is highly diverse and variable, with most of the full-length HEV genomes currently available in the GenBank database belonging to *Orthohepevirus A*. Based on several parameters, including host species, geographical origin, phylogenetic relationship, and disease outcome, *Orthohepevirus A* has been demonstrated to have at least eight different genotypes and 36 subtypes. To assign a viral subtype, at least three complete viral genomes that are phylogenetically and epidemiologically unrelated to previous strains must be available. Due to these stringent requirements for assigning subtypes, many divergent HEV strains have remained unassigned to date [19]. Genotypes 3 and 4 HEVs also exhibit notable genetic heterogeneity, as demonstrated by the zoonotic genotypes 3 and 4 HEVs, causing not only chronic HEV infections in immunocompromised patients but also extrahepatic diseases. In addition to the unique transmission patterns and clinical outcomes associated with different genotypes, HEV’s genetic variability has also been implicated in its resistance to antivirals. For example, multiple point mutations in the viral polymerase of genotype 3 HEV have been associated with ribavirin treatment failure in organ transplant recipients. Mutations Y1320H and G1634R are responsible for heightened viral fitness, while the K1383N mutation suppresses viral replication and increases their susceptibility to ribavirin [20,21,22].

## 4. Hepatitis E Virus Genome

**Figure 3:**
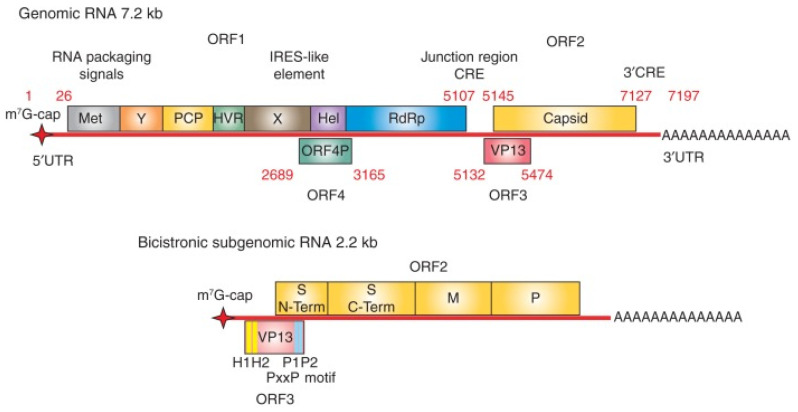
Organization of hepatitis E virus (HEV) genome. The genome of HEV is ~7.2 kb long comprising a single-stranded positive-sense RNA molecule. The genome contains a 7-methylguanosine RNA cap at the 5′ end and is polyadenylated at the 3′ terminus. There are three conserved open reading frames (ORFs) found in all known HEV strains: ORF1, ORF2, and ORF3. ORF1 encodes the nonstructural polyproteins with putative functional domains including methyltransferase (Met), Y domain, papain-like cysteine protease (PCP), hypervariable region (HVR), X domain, helicase (Hel), and RNA-dependent RNA polymerase (RdRp). ORF2 encodes the capsid structural protein. ORF3 encodes a multifunctional phosphoprotein (also known as VP13). ORF2 and ORF3 proteins are translated from a 2.2 kb long bicistronic subgenomic RNA. ORF 2 encodes the capsid protein, containing S, M, and P domains. PxxP is a highly conserved motif in ORF 3. In addition to these three ORFs, the genotype (gt) 1 HEV encodes an ORF4, which generates a protein from an internal ribosome entry site (IRES)-like element in response to endoplasmic reticulum stress (perturbation of homeostasis) (adapted from Kenney et al. [45] with permission).

Hepatitis E virus possesses a positive-sense, single-stranded RNA genome that is approximately 7.2 kb long and has a 7-methylguanosine RNA cap at the 5′ end and 3′ polyadenylated tail [24] (Figure 3). The viral genome typically encodes for three open reading frames (ORFs), namely ORF1, ORF2, and ORF3, but a fourth open reading frame (ORF4) (embedded within ORF1) present in only genotype 1 strains has also been described [25]. Open Reading Frame 1 is approximately 5 kb long located at the 5′ end, ORF2 is approximately 2 kb long at the 3′ end, and ORF3 is made up of 372 bases with its 5′ end overlapping ORF1 by just four nucleotides and its 3′ end overlapping ORF2 by 331 nucleotides [26,27]. In addition, HEV also produces a capped 2.2 kb bicistronic, sub-genomic RNA in infected cells that encodes for ORF2 and ORF3 [28,29]. The number of sub-genomic RNA copies is significantly higher than their genomic RNA counterparts in HEV-infected cells [30].

## 5. Hepatitis E Life Cycle and Host Interaction

**Figure 4:**
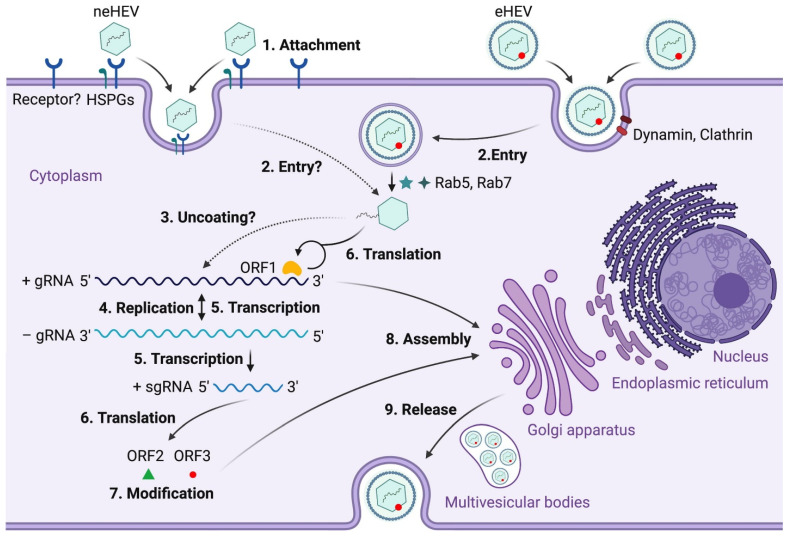
Hepatitis E virus (HEV) life cycle. (1) Non-enveloped hepatitis E viruses (neHEV) bind to heparan sulfate proteoglycans (HSPGs) on the surface of hepatocytes and enter through an unknown cellular receptor. (2) Quasi-enveloped HEV (eHEV) particles enter liver cells through dynamin-dependent, clathrin-mediated endocytosis, which requires the small GTPases Ras-related proteins Rab5 and Rab7. (3) Following the removal of the capsid protein through an unknown mechanism, the viral genomic RNA is released to the cytosol, serving as a template for ORF1 polyprotein translation as well as synthesizing a complementary negative-sense RNA template necessary for HEV replication (4). (5) The intermediate negative-sense RNA then serves as a template for transcription of both full-length genomic and subgenomic mRNAs (sgRNAs). (6) More ORF1 polyproteins are translated from the full-length genomic RNA, and the ORF2 capsid protein and ORF3 multifunctional protein are translated from the sgRNAs. (7) ORF2 and ORF3 undergo post-translational modifications such as glycosylation, phosphorylation, and palmitoylation. (8) ORF2 capsid protein self-assembles into virus-like particles (VLPs) and binds to newly synthesized positive-sense genomic RNA to form progeny HEV virions. (9) ORF3 regulates the host environment through interaction with a number of cellular proteins to promote viral replication and virion secretion (adapted from Wang et al. [41] with permission).

To date, information on the HEV life cycle and host interactions remains limited due to the lack of an efficient cell culture system to grow the virus. As a virus that is transmitted through the fecal–oral route, HEV first enters the host through the gastrointestinal tract where it starts replicating in intestinal epithelial cells before entering the bloodstream and finally reaching the liver [31] (Figure 4). Heparan sulfate proteoglycans (HSPGs) are required for HEV cellular binding, but the specific cellular receptor(s) necessary for the viral attachment is (are) yet to be identified [32]. Both quasi-enveloped HEV (eHEV) and non-enveloped HEV (neHEV) are thought to exploit unique virus-entry mechanisms. Quasi-enveloped HEV enters cells through a dynamin-dependent complex process involving small GTPases, the Ras-related proteins Rab5 and Rab7, and Niemann-Pick disease type C1-mediated lipid membrane degradation, but little is known about neHEV entry [33]. Following an unknown uncoating process of the HEV capsid, the viral genomic RNA directly serves as mRNA for ORF1 polyprotein translation, which produces several functional enzymes or domains. The viral replicase RdRp then synthesizes a complementary negative-sense RNA that serves as a template for HEV replication and transcription of the sub-genomic mRNA (sgRNA), which is responsible for translating the ORF2 protein and ORF3 protein. The negative-sense RNA has been detected in the livers and extrahepatic tissues of various animals experimentally infected with HEV [31]. The localization of ORF1 polyprotein to the endoplasmic reticulum (ER) membranes suggests that the ER may be the site of HEV replication [34,35]. Open reading frame 2 is produced as both a soluble, secreted glycosylated protein and a capsid-associated form. The former acts mainly as immune decoys while the latter assembles into virus-like particles and packages genomic RNA to progeny HEV virions [36]. Open reading frame 3 protein modulates the host environment, making it conducive for viral replication by interacting with several host cell proteins including microtubules [37,38,39]. Specifically, ORF3 protein binds to tumor susceptibility gene 101 protein (TSG101), a key factor involved in the transport (ESCRT) pathway, thereby facilitating the budding of nascent virions into multivesicular bodies [40]. The detailed structural and molecular functions of ORFs 1–3 have been extensively reviewed elsewhere [41]. The multivesicular bodies then fuse with the hepatocyte plasma membrane and the enveloped virions are released into the bloodstream while the quasi-enveloped virions are released into the bile duct, where they are degraded by bile salts [42]. It is important to note that the specific mechanisms involved in several steps of the HEV life cycle remain poorly understood due to the lack of efficient cell lines to propagate the virus. This, in turn, has slowed the progress in our understanding of HEV pathophysiology and diagnosis. While in vitro studies reporting HEV propagation in primary cell lines indicated inefficient viral replication, the detection of HEV in extrahepatic tissues of infected hosts offers the possibility of its propagation in cell lines derived from these tissues [31]. The development of efficient cell lines that support efficient HEV replication will undoubtedly increase the understanding of the virus’s life cycle, thereby providing better platforms for developing HEV-specific antiviral therapeutics [41,43,44].

## 6. Diagnosis of HEV Infection

### 6.1. Laboratory Diagnosis

After HEV exposure and infection, the virus incubates for 15 to 60 days [46]. Three weeks after infection, HEV RNA becomes detectable in both stool and blood [46]. The presence of HEV RNA shortly precedes clinical symptom onset, which is followed by biochemical marker elevation. Immunoglobulin M (IgM) antibodies develop at four weeks after infection, and IgG antibodies appear a few days later [47]. Viremia continues for three to six weeks; viral shedding into stool can persist for four to six weeks [48,49]. Immunoglobulin M antibodies are often present for three to four months, while IgG antibodies are present for years after primary HEV infection [50].

### 6.2. Molecular Testing

Molecular testing relies on nucleic acid amplification technology (NAAT)-based assays for the detection of HEV RNA. These assays use reverse transcription polymerase chain reaction (PCR) for RNA identification and amplification [51,52]. Conventional assays target HEV-conserved regions of ORF2 and ORF3 to detect all four genotypes of HEV [53]. Polymorphisms have resulted in false negatives, but the addition of a minor groove binding modification minimizes this risk through increased sensitivity [54]. 

Molecular tests detect the presence of HEV RNA in blood or stool, and this is indicative of active HEV infection. If the presence of HEV RNA persists for at least three months, a diagnosis of chronic hepatitis E can be made. Chronic infections predominately occur in immunocompromised patients, and these patients should be screened with molecular testing rather than antibody testing for HEV, since antibodies are often undetectable. Therefore, HEV RNA detection is the optimal method for the detection of HEV infection in these patients. 

HEV RNA can also be used to assess the viral load. This assessment is important in monitoring therapeutic responses. If the HEV RNA viral load is undetectable, it indicates successful treatment. However, the lack of a reduction or limitation in the viral RNA can indicate treatment failure and the need for additional treatment. Furthermore, the recurrence of viral RNA can indicate relapses in immunocompromised patients. 

### 6.3. Antibody Assays

Acute HEV infection can be diagnosed by the presence of anti-HEV antibodies. Immunoglobulin M antibodies develop shortly after clinical signs of infection or four weeks after exposure. Immunoglobulin G antibodies become positive a few days after IgM but will remain present for years rather than months. This serologic testing uses enzyme immunoassays in combination with HEV NAAT to detect antibody presence. Anti-HEV immunoglobulin A (IgA) testing has also been used with HEV assays. The positivity of anti-HEV IgM and IgA reportedly increases the sensitivity of testing, and in a study with 60 patients with HEV IgM and IgA positive tests, all patients were also positive for HEV RNA [55]. 

The negative features with this methodology include its unreliability in patients who are unable to produce antibodies, and patients on chronic immunosuppression are not good candidates for this testing [56]. In addition, antibody assays have a high degree of Epstein–Barr virus (EBV) and cytomegalovirus (CMV) cross-reactivity. In HEV IgM-positive samples, 33.3% and 24.2% of samples were also positive for EBV and CMV, respectively [56]. 

### 6.4. Antigen Assays

The detection of HEV using antigen detection with enzyme immunoassays can be used in both acute and chronic cases. The positivity rates of these tests were lower than reverse transcription polymerase chain reaction (rt-PCR) in both sera and stool with rates of 55% for sera and 76% for stool [29]. Enzyme immunoassays can remain elevated for multiple weeks in stool testing after HEV RNA becomes negative, and, therefore, HEV antigen detection does not directly correlate with the presence of infectious HEV virions [29]. 

### 6.5. Immunochemistry

Immunochemistry for the HEV ORF2 protein can be used to diagnose hepatitis E. Open reading frames 1 and 3 were both detectable in cells. However, they were not always unequivocally traceable or detectable [57]. 

## 7. Clinical Manifestations

### Hepatic Infection

Acute HEV infections present as an asymptomatic infection in the majority of patients. The frequency of symptomatic infections ranges from 5% to 30%, with the differences depending on the HEV genotype [58]. Hepatitis E virus genotypes 1 and 2 are the predominant genotypes in Asia, and these genotypes have a higher incidence of symptomatic presentations, at approximately 16% [59]. Hepatitis E virus genotype 3 is the predominant genotype in Europe and the United States [60]. The seroprevalence of genotype 3 in the United States is 6.1% [61]. HEV genotype 4 has been documented in Asia and more recently Europe but is not the most prevalent genotype in either location [62,63]. Both genotypes 3 and 4 have lower rates of symptomatic presentation, at 5% or less [60].

The typical time course of infection consists of incubation for 3 to 8 weeks following exposure. Symptomatic individuals will then develop a prodromal phase with malaise, fever, nausea, vomiting, and pruritis [64]. These symptoms last for one week with a subsequent icteric phase with dark urine and jaundice. Liver function tests peak approximately one week after prodromal symptom onset or 6 weeks from exposure in asymptomatic patients [64]. In immunocompetent patients, viremia will be cleared without further treatment. Liver function tests, including aspartate transaminase (AST), alanine transaminase (ALT), bilirubin, and international normalized ratio (INR), should be monitored for the resolution of infection. Patients will develop immunity against HEV after the clearance of acute infection. However, re-infection is still possible, but the risk of symptomatic hepatitis is decreased. 

Although HEV is typically cleared without difficulty, a small subset of patients will develop acute liver failure (ALF). With HEV genotypes 1 and 2 infections, 1% of symptomatic patients will progress to ALF [65]. This accounts for 40% of cases of ALF in South Asia and has an associated mortality rate of 40–60% [66,67]. Progression to ALF in HEV 3 is rare. However, one German study did find that in 80 cases of ALF, 10% of patients were positive for HEV RNA, indicating that HEV may be a more prevalent cause of ALF than previously thought [68].

In acute HEV infections in which patients are unable to clear HEV viremia, chronic HEV infection can develop. This has been defined as HEV viral replication for greater than 6 months and has been documented only with HEV genotypes 3 and 4 [13,69]. Patients at risk for chronic HEV include patients with HIV, organ transplantation, and rheumatologic disorders on high levels of immunosuppression [70]. Nucleic acid amplification tests are needed for diagnosis since these patients may not produce IgG or IgM antibodies in response to infections [70]. In a study of 85 organ transplant patients with HEV, one-third were symptomatic and the remainder had abnormal liver tests [70]. These HEV infections may progress into chronic cases in up to 50% of cases [71]. Undertreated prolonged chronic infections can progress to liver fibrosis and death [69]. These cases of liver fibrosis have been documented only with HEV genotype 3 [13]. The liver can recover from fibrosis secondary to HEV if treatment is initiated and viremia is cleared [72]. 

Specific patient groups seem to be at higher risk for HEV infections. In developed countries, HEV genotypes 3 and 4 infections tend to occur in older males with a 3:1 male-to-female ratio and at a median age of 63 [73]. It has been suggested that this population is at increased risk for symptomatic presentation as they typically have a higher prevalence of hepatic steatosis and fibrosis. In addition, patients with a high risk of underlying liver disease secondary to heavy alcohol use and diabetes are identified more frequently in HEV studies [74]. Hepatitis E virus likewise targets patients with known cirrhosis. In European countries, HEV accounts for 3% of cases of cirrhotic decompensation [75]. The mortality associated with decompensation itself was comparable to the mortality associated with other etiologies of cirrhotic decompensation. The mortality at 180 days in patients with HEV and cirrhosis was 22%, which is significantly higher than HEV alone at 3% [75]. 

In countries with a high prevalence of HEV genotypes 1 and 2, pregnant women are at a high risk for serious infection from HEV. Pregnant patients have a ten-fold higher risk of ALF with HEV infection compared to non-pregnant patients [76]. Mortality in pregnant and non-pregnant patients with ALF secondary to HEV is not significantly different, but pregnant patients with HEV do have increased rates of preterm labor and fetal loss [67,77]. These complications have been reported in HEV genotypes 3 and 4 as well, but this information is limited to case reports. 

## 8. Extrahepatic Complications

Patients infected with HEV have a range of presentations, including no symptoms, acute hepatitis, and fulminant hepatitis. Some patients also develop extrahepatic manifestations, including neurological, hematological, and renal disorders, that can occur in both acute and chronic HEV infections. The pathogenesis of extrahepatic manifestations of HEV infection is not well understood [78]. The hypotheses include the development of host systemic immune responses in extrahepatic organs after HEV infection and HEV viral replication in non-hepatic tissues, supported by the evidence of HEV found in human placenta, breast milk, urine, and neuronal cells [78].

### 8.1. Neurologic Complications

Hepatitis E virus has been associated with several neurologic presentations, and 16.5% of patients in France infected with HEV have reported neurologic symptoms [79]. These cases have been documented in Europe, Asia, and the United States [80]. In general, HEV genotypes 1 and 3 are associated with neurologic manifestations [80]. The neurologic syndromes include Guillain–Barré syndrome, neuralgic amyotrophy, encephalitis, myelitis, and Bell’s palsy. The most frequent complications are Guillain–Barré syndrome and neuralgic amyotrophy [80,81]. 

Neuralgic amyotrophy is an acute and painful mononeuropathy/polyneuropathy in the upper extremities with motor weakness and sensory loss. Patients usually complain of shoulder pain followed by flaccid weakness and muscle wasting. The underlying pathophysiology remains uncertain, but 10% of recorded cases in the UK and Netherlands tested positive for acute HEV [82]. In 80% of HEV-positive cases, patients presented with bilateral asymmetric involvement of the brachial plexus [83]. Therefore, the presentation of neuralgic amyotrophy with bilateral involvement should prompt consideration of a possible HEV infection. In addition, neuralgic amyotrophy with phrenic nerve damage was present in many HEV-positive neuralgic amyotrophy cases [83]. 

Guillain–Barré syndrome is a rapidly ascending flaccid paralysis with numbness, secondary to the autoimmune destruction of peripheral nerves. In the Netherlands, Belgium, and France, 5 to 8% of cases were preceded by an acute HEV infection [84,85]. One Bangladesh case–control study found that anti-HEV IgM was more frequently detected in patients with Guillain–Barré syndrome than with other neurologic conditions, with an incidence of anti-HEV IgM positivity in 11% of cases [86]. Therefore, a definite association has been established between Guillain–Barré syndrome and HEV infection. 

Less-frequent neurologic manifestations of HEV include encephalitis and myelitis. These were reported in a small case series in Europe, Asia, and the USA. Both central and peripheral neurologic complaints were reported, with ataxia the most common complaint. Five of 12 cases were reported in solid-organ transplant recipients and involved HEV genotype 3 [80], and two cases died [80]. Other reported neurologic manifestations include Bell’s palsy, mononeuritis multiplex, peripheral neuropathy, and myositis. 

### 8.2. Hematologic Complications

Hepatitis E virus infections have been associated with glucose-6-phosphate dehydrogenase (G6PD) deficiency, autoimmune hemolytic anemia, and thrombocytopenia. Glucose-6-phosphate dehydrogenase deficiency is the most common hematologic manifestation in acute HEV infections, and one study found that G6PD occurred in 70% of cases with acute HEV hepatitis [87]. Autoimmune hemolytic anemia presents with spherocytes on peripheral smear, laboratory findings indicative of hemolysis, and direct antiglobulin test (DAT) antigen positivity in 85% of cases and occurred in 23% of acute HEV hepatitis cases [87]. Thrombocytopenia has also occurred during acute HEV infection. There is still uncertainty about the underlying pathophysiology of thrombocytopenia and proposed mechanisms include hypersplenism, reduced hepatic thrombopoietin production, bone marrow suppression, and anti-platelet antibodies [88]. 

Other less-common hematologic manifestations include pancytopenia, cryoglobulinemia, and lymphoproliferative disorders. Pancytopenia and aplastic anemias occurred two to three months after acute infections [89]. This presentation has been found with various forms of viral hepatitis and is not isolated to HEV. Cryoglobulinemia has occurred in acute and chronic cases of HEV. These patients present with arthralgia, rash, and myalgia with symptom resolution after treatment of HEV infection [90]. Documented lymphoproliferative disorders associated with HEV include monoclonal gammopathy of undetermined significance and CD30-positive cutaneous T-cell lymphoproliferative disorder [91,92]. Consideration of HEV in the workup of hematologic disorders is recommended since treatment of HEV infection can lead to the resolution of these disorders. 

### 8.3. Renal Complications

The most common renal manifestations with HEV infection are membranoproliferative glomerulonephritis (MPGN), IgA nephropathy, and cryoglobulinemia. Membranoproliferative glomerulonephritis and IgA nephropathy have occurred mostly during acute HEV genotype 3 infections [93]. The overall clinical significance remains minimal. However, patients had an average of five-point decrease in glomerular filtration rate when affected by these disorders [93]. Cryoglobulinemia has occurred in both acute and chronic cases of HEV in solid-organ transplant patients. In HEV-positive patients with solid-organ transplants, 36% of acute cases and 52% of chronic cases developed cryoglobulinemia [90]. The symptoms resolved after treatment of the HEV infection in both acute and chronic cases. 

Other extrahepatic manifestations in other organs include pancreatitis, thyroiditis, and myocarditis [94]. Overall, neurologic complications are the most common extrahepatic manifestation of HEV infection [95]. Immunological dysregulation might have an important role in developing extrahepatic manifestations, and more study on the pathogenesis of these complications is needed.

## 9. Chronic Hepatitis E Virus Infection

Chronic HEV infection was first reported in 2018 [13]. Chronic infection is more common in immunocompromised patients with solid-organ transplantation, hematologic disorders or malignancy, and HIV infection who are unable to spontaneously clear the virus. This leads to persistent infection, defined as positive HEV RNA longer than 3 months from the onset of acute infection. Hepatitis E virus genotypes 3 and 4 are the most common causes of chronic HEV infection. Treatment of chronic HEV infection has been studied using ribavirin, pegylated interferon-alpha, and sofosbuvir.

### 9.1. Pathogenesis of Chronic HEV Infection

The pathogenesis of chronic hepatitis E infection is not well understood, and studies are limited to in vitro models. The adaptive immune response may contribute to the pathogenesis of these infections [96], and several studies have reported correlations between T-cell responses and persistent HEV infection. Suneetha compared the T-cell response in HEV infection in 38 individuals: organ transplant recipients (*n* = 19) and healthy controls (*n* = 19) [96]. The study focused on T-cell responses after peptide proteins that stimulate T-cells were administered; the experiments then measured T-cell responses based on the proliferation and cytokine production of CD4^+^ and CD8^+^ T-cells. This study reported that in patients with chronic HEV infection the T-cell responses were absent but became detectable after viral clearance. The authors suggested that improving adaptive cellular immunity might decrease the development of persistent HEV infection. This study also found that T-cells produce interferon (IFN)-gamma after stimulation, which should suppress virus replication. The peptides used in this study were derived from genotype 1, whereas chronic HEV infection is usually caused by HEV genotype 3. Consequently, the role of immune responses by T-cells in HEV infections and in re-infections deserves further study.

### 9.2. Chronic Hepatitis E in Solid-Organ Transplant Recipients

Before 2008, HEV was thought to cause only acute viral hepatitis, but in 2008, Kamar first reported cases of chronic HEV infection in solid-organ transplant recipients [13]. The prevalence of HEV in solid-organ transplant patients is approximately 20% [97]. The prevalence of HEV IgG antibodies is similar to the general population, suggesting that the frequency of the acute infection of HEV in solid-organ transplant recipients is relatively low [98,99]. The solid-organ transplantations associated with these infections include lung, liver, and kidney transplantation; information on chronic infection in heart transplantation patients has been limited to a case series or case report [71,100]. 

Kamar reported 14 solid-organ transplant recipients (three liver, nine kidney, and two kidney and pancreas recipients) who developed acute HEV infection [13]. Of these 14 patients, seven patients were symptomatic and developed acute viral hepatitis symptoms, i.e., weight loss and jaundice; the other seven patients had abnormal liver enzymes. Eight patients (57.1%) developed chronic HEV infection, defined by the persistence of infection 6 months after the end of the acute phase. In patients with chronic HEV infection in this study, the mean duration of the elevated liver enzymes and HEV RNA in stool was 15 months after the end of the acute phase of HEV infection. Of note, all 14 patients were being treated with conventional doses of immunosuppressive medication. Six of eight patients (75%) with chronic HEV infection had a liver biopsy, which confirmed chronic viral infection characterized by liver fibrosis and portal hepatitis. The patients whose disease courses progressed to chronic HEV infection had significantly lower CD2, CD3, and CD4 lymphocyte levels at baseline, suggesting that the T-cell response has an important role in HEV clearance. Another study reported that the factors associated with chronic HEV infection were tacrolimus treatment, low platelet counts, coinfection with hepatitis B virus, and hepatocellular carcinoma [97]. 

Abnormal laboratory tests with elevated liver enzymes but normal serum bilirubin levels often develop in asymptomatic patients who have HEV infection [101]. Most solid-organ transplant recipients will have mild elevation of liver enzymes regardless of symptoms. The anti-HEV IgM and IgG levels may remain negative due to reduced production of HEV-specific antibodies. Therefore, HEV RNA detection with the PCR technique is recommended as a screening test for HEV infection in organ transplant recipients [102]. 

Elevation of liver enzymes in solid-organ transplantation is often misinterpreted as graft versus host disease, especially in liver transplantation, in which almost every patient will have an elevation of liver enzymes [103]. This makes it difficult to distinguish post-liver-transplant patients with viral infection from transplant patients with graft versus host disease (graft rejection), vascular complications, biliary complications, or drug-induced liver injury [102,103,104]. These misinterpretations might lead to a lower number of reported chronic HEV infection cases in solid-organ transplant patients who were found to have elevated liver enzymes.

### 9.3. Liver Transplantation

Hepatitis E virus infection is an important cause of graft hepatitis in liver transplantation patients [104]. The annual incidence of HEV infection in post-liver-transplantation patients who were previously seronegative prior to transplantation is approximately 2.1% [99]. Buffaz studied the prevalence and incidence of chronic HEV infection in liver transplant recipients and recruited 206 pediatric and adult patients who underwent liver transplantations. The study reported that three patients (1.45%) developed chronic hepatitis; the mean HEV IgG seroprevalence was 29% pre-transplantation and 28% post-transplantation. This study also reported two cases of re-infection with HEV [105]. Liver transplant patients who are infected with HEV have an increased risk of developing cirrhosis [98].

Pischke et al. studied the association of chronic HEV infection and graft hepatitis following liver transplantation [106]. The study recruited 493 patients; 226 were liver transplant recipients, 129 were non-transplanted patients with chronic liver disease, and 108 were healthy controls. These authors categorized liver transplant recipients into two groups according to the clinical evidence of graft hepatitis [106]. One hundred fifty-six patients (69.0%) did not have evidence of graft hepatitis, and 70 patients had elevated alanine transaminase (ALT), aspartate transaminase (AST), gamma-glutamyl transpeptidase (GGT), or bilirubin levels (at least twice the upper level of normal) and had a liver biopsy. Out of the graft hepatitis group (70 patients), 33 patients had graft rejection, seven had graft hepatitis, and 13 patients had other viruses (EBV, herpes) as a cause of graft hepatitis. No definite cause of graft hepatitis was found in the other 27 patients. This study used both anti-HEV IgG and HEV RNA (only one needed to be positive) to establish the diagnosis of chronic HEV infection. In liver transplantation recipients (226 patients), the prevalence of de novo infection of chronic HEV was 1.6%, including two out of 156 in the group without clinical evidence of graft hepatitis (group 1) and two out of 70 in the group with clinical evidence of graft hepatitis (group 2). This study suggested that all patients with elevated liver enzymes should be tested for HEV RNA unless other obvious reasons explain the cause of hepatitis.

### 9.4. Kidney Transplantation and Heart Transplantation

A Japanese nationwide survey study reported that the prevalence of hepatitis E in patients who tested positive for anti-HEV IgG in kidney transplant and heart transplant recipients was 4.1% (103 in 2526) in kidney transplant recipients and 7.1% (7 in 99) in heart transplant recipients, respectively [107]. However, HEV RNA was positive in 12 patients (11 kidney transplant recipients and one heart transplant recipient) with a prevalence of 0.4% in kidney transplant patients and 1.0% in heart transplant patients. All patients who tested positive for HEV in this study were infected with HEV genotype 3. Out of the 12 positive HEV RNA patients, five patients had chronic infection (four kidney transplants (36.4%) and one heart transplant (100%)). The risk factor for HEV infection in this study was suspected to be foodborne, i.e., from eating undercooked pig, deer, and boar.

Chronic HEV infection in kidney transplant recipients might result in a rapid progression of liver diseases, resulting in liver decompensation [69]. Kamar reported two cases of kidney transplantation recipients who developed decompensated liver failure within 2 months after the diagnosis of the acute phase of HEV infection, suggesting that patients can develop acute liver failure even in the acute phase (before developing chronic infection at 6 months) [69].

Pischke studied the prevalence of HEV infection in 274 heart transplant recipients and 537 healthy controls and reported a higher prevalence in heart transplant recipients (31 out of 274, 11.3%) compared to healthy control patients (11 out of 537, 2%) with a *p*-value < 0.001 [71]. Of these 31 heart transplant recipients, 11 had infections prior to transplantation. (One patient’s pre-transplant serum was not available.) Nineteen heart transplant recipients (6.9%) had HEV infections after transplantation. Four patients (1.5%) developed chronic HEV infections. This study suggested that chronic HEV infection should be in the differential diagnoses in patients who have undergone heart transplantation and have an elevations of ALT above 200 IU/L. Three patients who developed chronic HEV infections were found to have liver fibrosis, cirrhosis, progressive liver disease, and hepatic decompensation.

### 9.5. Hematopoietic Stem Cell Transplantation

There are several reported studies of hematopoietic stem cell transplantation patients with chronic HEV [108]. Versluis reported the prevalence of HEV infection in allogenic hematopoietic stem cell transplantation was 2.4% (eight out of 328 allogenic hematopoietic stem cell transplantation recipients); five out of 328 (1.5%) developed chronic HEV infection [108]. This study recommended screening for HEV infection before hematopoietic stem cell transplantation, and the diagnosis of HEV infection should be considered in hematopoietic stem cell transplantation recipients who have liver enzyme abnormalities.

## 10. Chronic HEV Infection in Immunocompromised Patients

### 10.1. Human Immunodeficiency Virus Infection

The prevalence of HEV in HIV-infected patients is reported to be 5–21% [109]. These patients are considered highly sensitive to HEV infection [110], based on a case report and case series of chronic hepatitis E in patients with HIV infection [111]. The reported prevalence of chronic HEV infection in HIV is approximately 0 to 0.5% [110]. Studies have reported that HIV patients who developed chronic infections often had CD4 cell counts of fewer than 200 cells/mm^3^ [110,112]. Neukam reported two cases of chronic HEV who were severely immunocompromised with CD4 < 100 cells/mm^3^ [112]. These two patients did not have symptoms but had elevated ALT levels and liver stiffness on ultrasound. These patients subsequently developed cirrhosis within 3 years; they were treated with ribavirin monotherapy, which cleared the infection.

### 10.2. Hematologic Malignancy

Chronic HEV infection has been reported in patients with hematologic malignancy, including non-Hodgkin’s lymphoma, lymphoplasmacytic lymphoma, myelodysplastic syndrome, and diffuse large B-cell lymphoma [113,114,115]. A case of reactivation of HEV infection in a patient with acute lymphoblastic leukemia has been reported [116]. That patient had acute hepatitis E prior to stem cell transplantation, and 14 weeks after stem cell transplantation, HEV viremia was found along with an increased viral load and increased liver enzymes. Immunosuppressive therapy can suppress T-cells and lead to persistent viremia in patients who have received chemotherapy. Tamura studied two patients with T-cell lymphoma who had chronic HEV infections during chemotherapy transmitted by blood transfusion; these two patients had persistent HEV RNA in the blood and did not develop HEV antibodies at a 6 month follow-up, possibly secondary to immunosuppressive therapy [117].

### 10.3. Rheumatological Diseases

A European retrospective multi-center case series recruited 21 patients in a rheumatology and internal medicine clinic who tested positive for anti-HEV IgG and studied the disease course, treatment, and outcome after the diagnosis of HEV [118]. The underlying diseases in these patients included rheumatoid arthritis, psoriatic arthritis, other variants of chronic arthritis, primary immunodeficiency, systemic granulomatosis, lupus erythematosus, Erdheim–Chester disease, and retroperitoneal fibrosis. Seven out of 21 patients (33.3%) developed chronic HEV infections, defined as persistent HEV infection longer than 3 months, and five patients (24%) had infections last longer than 6 months. Of these five patients with HEV infections, four patients tested positive for genotypes 3c and 3f, and one patient tested positive for genotype 1, with the HEV presumably imported from another country. The ages of these patients ranged from 29 to 75 years; the underlying diseases were CVID (one was lost to follow-up), RA (two), primary immunodeficiency (one), granulomatosis (one), retroperitoneal fibrosis (one), and undefined CD4 disturbance (one). Five patients received ribavirin for treatment; one patient had rheumatoid arthritis, and viremia persisted for 16 weeks after the discontinuation of immunosuppression (Abatacept). This study demonstrated that chronic HEV infection occurs not only in transplant recipients and HIV patients but also in patients with rheumatologic disorders. 

Kounis retrospectively studied 488 patients with inflammatory bowel disease (IBD) who were being treated with immunomodulator therapy and reported three cases of HEV IgM-positive patients; none had a positive HEV RNA test, indicating no cases of chronic HEV infection [119]. This study does not support screening for HEV in patients with IBD who are receiving immunosuppressive therapy. 

## 11. Treatment of Chronic HEV Infection

Unlike healthy adults, immunocompromised patients with HEV infection can develop chronic persistent infection that can lead to cirrhosis and death [120]. The management of chronic HEV in these patients, especially patients with solid-organ transplantation, remains difficult and may require altering the immunosuppressive regimens while treating the HEV infection. Decreasing the immunosuppressant drugs targeting T-cell responses can help eliminate HEV in patients with chronic infections. The European Association for the Study of the Liver (EASL) has suggested that reducing immunosuppressive drug doses, especially drugs targeting T-cells, could be a useful initial therapeutic option [4].

Wang studied the effect of multiple immunosuppressive drugs on viral replication in vitro in host cells infected with HEV genotype 3. The study suggested that calcineurin inhibitors (particularly cyclosporin A) might lead to a persistent viral infection. The study concluded that corticosteroids do not have a direct effect on HEV replication; however, cyclosporin A had a dose-dependent effect in promoting HEV replication. Mycophenolic acid inhibited HEV replication, and the combination of mycophenolic acid with ribavirin had extended antiviral activity.

Currently, the EASL recommends ribavirin, pegylated-interferon-alpha, or combination therapy as a standard treatment in patients with chronic HEV infections. However, the dose and duration for optimal therapy are still unknown [4]. In patients who have persistent HEV infections despite receiving treatment for 3 months, additional monotherapy with ribavirin is recommended for another 3 months (therapy for 6 months total).

### 11.1. Ribavirin

Ribavirin is an antiviral medication used to treat RSV infection and hepatitis C. It is a guanosine analog and inhibits viral RNA synthesis. Kamar performed a multi-center study on the efficacy of ribavirin as a monotherapy in treating acute and chronic HEV infection [121]. The study recruited 59 patients with solid-organ transplants (37 kidney transplants, ten liver transplants, five heart transplants, five kidneys and pancreas transplants, and two lung transplants). This study reported that the median dose of ribavirin given was 600 mg/day (8.1 mg/kg/day), with a median duration of 3 months (range 1 to 18 months). After treatment, 46 patients (78%) achieved sustained virologic responses (SVR), defined as an undetectable serum HEV RNA level at 6 months after the ribavirin was completed. This study also reported that a higher lymphocyte count before the initiation of the treatment with ribavirin may be associated with SVR [121]. Abravanel conducted a retrospective study on the efficacy of ribavirin in chronic HEV infection in 24 solid-organ transplant recipients (16 kidney transplants, five liver transplants, two heart transplants, and one lung transplant) [122]. Before the treatment with ribavirin, Abravanel and colleagues checked the HEV RNA in both serum and stool of the patients included in the study. The treatment included ribavirin for 3 months with a dose range of 200 to 800 mg/day. After the ribavirin monotherapy, 15 patients (62.5%) achieved SVR; nine patients (37.5%) had a relapse. In the relapse group, six patients (66.6%) had positive stool HEV RNA 3 months after treatment with ribavirin, while none of the SVR group had positive stool HEV RNA. After 3 months, both groups had no HEV RNA detected in their plasma. This study suggested testing for HEV RNA in both serum and stool samples at the end of treatment to make sure eradication occurs and to prevent relapses. 

A recent meta-analysis included 34 studies with 582 patients and studied the effectiveness in viral clearance with reductions of immunotherapy, ribavirin, and pegylated-interferon-alpha [123]. The study showed that a reduction of immunosuppressive therapy in 174 patients produced a SVR in 55 patients (32%), while treatment with ribavirin produced a SVR in 301 from 395 patients (76%); a random-effects model estimated that treatment with ribavirin had a pooled rate of SVR of 78% (95 CI: 72–84%), pegylated interferon-alpha produced a SVR in 11 of 13 patients (84%). Relapses occurred in 18% of the patients. The side effects from treatment with ribavirin included anemia requiring blood transfusion or erythropoietin or dose reduction in 37% of patients. A second treatment attempt with ribavirin led to an SVR in 39 out of 51 (76%) of the patients [123]. Another study showed that 10% of chronic HEV patients may have no response to ribavirin or remain viremic despite long-term treatment with ribavirin [124]. A recent multi-center study showed that the SVR after ribavirin monotherapy was 81.2% and increased to 89.8% in patients offered a second course of ribavirin [125].

### 11.2. Side Effects of Ribavirin

Despite being the most-recommended medical therapy for chronic HEV infection, ribavirin has side effects due to bone marrow toxicity [126]. Based on a meta-analysis, side effects from ribavirin occurred in 122 out of 395 patients (30.9%) [123]. These side effects included anemia, pancytopenia, psychiatric symptoms, and decreases in renal function; anemia was the most common side effect of ribavirin and led to multiple transfusions and erythropoietin injections in more than half the patients [127]. Ribavirin dose adjustment is often needed.

### 11.3. Ribavirin Treatment Failure

Low et al. studied the treatment outcome of ribavirin treatment and the risk of ribavirin treatment failure in ten patients who underwent solid-organ transplants and had chronic HEV infections in Singapore [128]. Of these ten patients, five had kidney transplants, four had liver transplants, and one had a bone marrow transplant. Nine patients were infected with HEV genotype 3 and resided in Singapore; one patient from another country had HEV genotype 7. The study outcome included nine patients at 3 months follow-up after ribavirin therapy, and two patients had persistent viremia. Of these two patients, one achieved SVR after a reduction in the ribavirin dose due to the side effect of symptomatic anemia; the other patient was noncompliant with treatment. Four patients developed recurrent HEV infection; all four were kidney transplant patients despite having SVRs at the end of therapy. Therefore, the overall failure rate of achieving SVR after 12 weeks of ribavirin therapy in this study was 66.67% (six patients out of nine). All four patients with recurrent infection had transient transaminitis, and reinfection with HEV could not be excluded. All four patients achieved SVR after the second course of ribavirin therapy for 12–16 weeks. This study suggested that kidney transplantation might influence the SVR rate. This study is limited by the sample size and the initiation of ribavirin before 3 months after the detection of HEV RNA, which means that not all patients might have developed chronic infection requiring treatment.

### 11.4. Pegylated Interferon-α

Pegylated interferon-α (PEG-IFN-α) has been used for over thirty years in patients with chronic hepatitis B infection [129]; interferons are cytokines that modulate the host defenses and inhibit viral replication. However, the use of this medication in chronic HEV infection has not been well-studied. It is important to note that PEG-IFN-α is contraindicated in lung, heart, renal, and pancreas transplant recipients due to the risk of organ rejection [130]. However, it is thought to be relatively safe in liver transplant patients, but there is no strong evidence on whether PEG-IFN-α is effective in treating chronic HEV infection in liver transplant patients. Although it is not recommended to use in kidney transplant patients, Kamar reported a case of chronic HEV in a kidney transplant patient who required hemodialysis with a 3-month course of PEG-IFN-α at a weekly dose of 135 μg that resulted in SVR [131]. Haagsma et al. described two liver transplant recipients with chronic HEV infections with durations of infection of 9 years and 9 months and reported success with PEG-IFN-α-2b as a monotherapy in treating chronic HEV infection [132]. A study of PEG-IFN-α and SVR in chronic HEV infection showed approximately 25% (two out of eight patients) achieved SVR [130].

### 11.5. Sofosbuvir

Given the side effects of the standard recommended therapy with ribavirin, the use of sofosbuvir in chronic HEV infection has been proposed. Sofosbuvir inhibits viral RNA synthesis, has been used in the treatment for chronic hepatitis C virus (HCV) infection, and has an efficacy of 95% (95% CI: 91–98%) [133]. To date, studies on the use of sofosbuvir as a monotherapy in chronic HEV infection are limited with most studies focusing on an adjunctive use with ribavirin. In in vitro studies, sofosbuvir inhibits HEV genotype 3 replication and has an additive effect when given with ribavirin [134]. However, in comparison to the strong antiviral effect of sofosbuvir on HCV in vitro, sofosbuvir had only a moderate effect on HEV. Cornberg performed a pilot study focusing on the effectiveness of sofosbuvir as a monotherapy in treating chronic HEV [135] and recruited nine patients. The mean age was 44.0 ± 13.7; the mean duration of infection was 29.1 ± 29.1 months. All patients, including heart/kidney/stem cell/pancreas transplants, were infected with HEV genotype 3. The study reported that no patient had undetectable HEV RNA at the end of therapy (12 weeks of sofosbuvir). However, five patients had reductions in HEV RNA levels, which suggested a modest antiviral effect of sofosbuvir in the treatment of chronic HEV infection [135]. A study of combination therapy of sofosbuvir and ribavirin after the failure of ribavirin monotherapy in three solid-organ transplant patients (one heart transplant and two lung transplants) with sofosbuvir 400 mg/day reported failure in achieving SVR in all cases [136].

In summary, the first step in the management of immunocompromised patients with chronic HEV infection is to reduce the dose of immunosuppressive therapy before considering drug treatment [135]. Antiviral therapy is the second step, and ribavirin remains the best drug of choice for chronic HEV infection. Treatment with PEG-IFN-α can be considered in liver transplant patients who are refractory to ribavirin, but it cannot be used in heart and kidney transplant recipients given the high risk of graft rejection. 

## 12. Hepatitis E Vaccine

A recombinant vaccine for HEV, known as “Hecolin”, was first introduced in China in 2011 [2]. The vaccine has been approved by the Chinese FDA and has been available since 2012. However, it is not available in other countries, and no vaccine has been approved by the FDA in the United States. The vaccine is given in three separate doses at 0, 1, and 6 months. The efficacy of the HEV vaccine was reported to be 100% during the first 12 months and 86.6% at 4.5 years follow-up [137,138]. It is effective against genotype 4, but there are no data on cross-protection against other genotypes [139]. Hecolin has been used in pregnant women with little apparent risk or adverse effects in the mother and fetus. Data on the use of the vaccine in individuals younger than 16 are limited, and there have been few large-scale studies [140]. Given the high mortality rate in pregnant women and the risk of developing chronic HEV infection in an immunocompromised host, this vaccine should probably undergo more study, especially in highly endemic regions and during outbreaks. In addition, it needs evaluation in immunocompromised patients.

## 13. Conclusions

Hepatitis E virus has a worldwide distribution and is transmitted through a fecal–oral route in regions of the world with inadequate management of water supplies. It can also cause periodic epidemics during environmental events, such as flooding. In healthy adults, this virus usually causes asymptomatic hepatitis, but it can cause severe hepatitis in pregnant women. In patients with chronic illnesses and immunosuppression, chronic hepatitis can develop and progress to cirrhosis and death. Patients at risk for chronic infection include patients with organ transplantation, chronic immunosuppression, and HIV infection. Some patients develop neurologic complications such as Guillain–Barré syndrome, hematologic complications such as glucose-6-phosphate dehydrogenase deficiency, and renal complications such as membranoproliferative glomerulonephritis. The diagnosis of acute infection depends on the measurement of IgM antibodies and the detection of viral RNA in serum and stool specimens. The diagnosis of chronic infection depends on the measurement of IgG antibodies. Antiviral medications, such as ribavirin, sofosbuvir, and interferon, are used to treat patients with chronic infection. In addition, patients on chronic immunosuppressive medication should have the dose reduced if possible. Finally, clinicians throughout the world need to consider this virus when patients present with acute hepatitis, chronic hepatitis, or unexplained extrahepatic complications. Travel to endemic regions potentially places everyone at risk for this infection.

## Data Availability

Not applicable.
